# Bevacizumab Injection in Patients with Age-Related Macular Degeneration Associated with Poor Initial Visual Acuity

**DOI:** 10.1155/2012/861384

**Published:** 2011-11-29

**Authors:** Leila El Matri, Rym Bouraoui, Ahmed Chebil, Fedra Kort, Mejda Bouladi, Rym Limaiem, Hana Landoulsi

**Affiliations:** Department B of Ophthalmology, Hedi Rais Institute of Ophthalmology, Boulevard 9 Avril, Bab Saadoun Tunis 1006, Tunisia

## Abstract

*Purpose*. To evaluate functional and anatomic effects of intravitreal bevacizumab in patients with neovascular AMD and initial low visual acuity. *Methods*. Retrospective case series of 38 eyes with neovascular AMD and initial visual acuity of 20/200 or less, treated with intravitreal bevacizumab injection. *Results*. Mean followup was 14.1 months ±  7.1 (range: 5 to 24 months). Mean logMAR vision at baseline was 1.38 logMAR ±  0.33, at 6 months was 1.14 logMAR ±  0.37 (*P* = 0.001) and at 12 months was 1.22 logMar ±  0.33 (*P* = 0.004). Mean baseline central retinal thickness was 431 *μ*m ±  159.7 at 6 months was 293.43 *μ*m  ±  122.79 (*P* = 10^−4^) and at 12 months was 293.1 *μ*m  ±  130 (*P* = 0.004). Visual acuity improved in both patients with or without prior PDT treatment. *Conclusions*. Intravitreal bevacizumab injection may increase the chance of visual acuity gain in neovascular AMD even in cases with initial low visual acuity.

## 1. Introduction

Since the vascular endothelial growth factor (VEGF) has been implicated as a major angiogenic stimulus responsible for the formation of choroidal neovascularization (CNV) in age-related macular degeneration (AMD) drugs inhibiting the bioactivity of VEGF representing a new paradigm in the treatment of neovascular AMD [1]; MARINA (Minimally Classic/Occult Trial of the Anti-VEGF Antibody Ranibizumab in the Treatment of Neovascular Age-Related Macular Degeneration) [2] and ANCHOR (Anti-VEGF Antibody for the Treatment of Predominantly Classic Choroidal Neovascularization in Age-Related Macular Degeneration) [3] studies, both multicenter, randomized, double-masked trials, established the efficacy of ranibizumab (Lucentis) in improving visual acuity in patients with both classic and occult CNV. Both studies excluded individuals with very low visual acuity (range, 20/40–20/320 in the MARINA study and 20/80–20/120 in the ANCHOR study) and patients previously treated with photodynamic therapy. However, subgroup analysis of the MARINA trial noted that the improvement in patients with initial visual acuity of 20/160 or less is lower than patients with better initial visual acuity [4]. Therefore, there are limited data concerning the effect of anti-VEGF drugs in patient with neovascular AMD and initial low visual acuity. Bevacizumab (Avastin; Genentech, San Francisco, Calif, USA) is a full-length antibody against all VEGF-A isoforms which is approved for use in metastatic colon cancer and lately used for the treatment of AMD.

The purpose of the present study was to evaluate the functional and the anatomic effects of intravitreal bevacizumab in patients with wet AMD and initial low best corrected visual acuity (BCVA).

## 2. Materials and Methods

We conducted a retrospective consecutive case series of 38 patients (38 eyes) with neovascular AMD and initial low visual acuity in a referral centre for AMD patients in Tunisia from June 2006 to December 2009.

An ophthalmic examination was performed, including measurement of best-corrected visual acuity (BCVA) using the ETDRS visual acuity protocol, slit-lamp biomicroscopy, intraocular pressure (IOP) measurement and contact lens slit-lamp biomicroscopy, colour fundus photography, digital fluorescein angiography (FA; Imagenet; Topcon Corporation, Tokyo, Japan), indocyanine green angiography (ICGA), and optical coherence tomography (OCT) scanning (OTI, Toronto, Canada). The same experienced ophthalmologist performed all FA, and OCT evaluations. The data recorded included complains as scotoma, blurred vision and metamorphopsia, BVCA, lesion type on FA and central retinal thickness (CRT) on OCT.

The BCVA and OCT were performed at baseline for every 6 weeks. FA and ICGA were performed at baseline and every 3 months afterwards.

The following inclusion criteria were applied: (1) exudative AMD, (2) initial visual acuity of 20/200 or less, and (3) with or without prior treatment of CNV as photodynamic therapy or laser photocoagulation. The Exclusion criteria were (1) subretinal fibrosis involving central fovea and (2) presence of other ocular pathology reducing vision.

Patients with BCVA better than 20/200 before treatment, uncontrolled systemic arterial hypertension (blood pressure >180/110 mmHg), history of thromboembolic event, renal disease, and recent surgery were excluded.

All patients were treated at baseline with intravitreal bevacizumab (1.25 mg/0.05 mL). A standard protocol for intravitreal injections was followed, including the operative room, the use of topical 5% povidone-iodine, eyelid speculum, and postoperative topical antibiotic drops. They were asked to return the following day for assessment of IOP and signs of intraocular inflammation or infection. Whenever IOP exceeded 24 mmHg, patients were given topical medication to reduce the pressure.

Indications for retreatment by IVB were defined as persistent subretinal and/or intraretinal fluid on OCT for every 6 weeks. No repeat treatment was performed if cessation of dye leakage from the CNV was revealed in FA, as well as total resolution of the subretinal fluid on OCT.

For statistical analysis, ETDRS acuities were transformed to log-MAR units (logarithm of the minimum angle of resolution). Person correlation test, paired Wilcoxon test and chi-square test were performed using SPSS 14.0 for Windows. The paired Student's *t*-test was used to statistically evaluate changes in logMAR visual acuity and central retinal thickness at different time points. In all analyses, a *P* value <0.05 was considered to be statistically significant. 

## 3. Results

### 3.1. Baseline Characteristics

In the current study, 38 eyes of 38 patients were enrolled. The mean age of the patients (23 men and 14 women) was 73 years ± 6.24 (range 62–82 years). The mean followup was 14.1 months ± 7.1 (range 5–24 months).

Ten patients (26.3%) had a history of photodynamic therapy (PDT). CNV was minimally classic in 11 eyes (28.9%), occult in 16 eyes (42.1%), predominantly classic in 1 eye (2.6%), and classic in 10 eyes (26%). A pigment epithelial detachment was present in 4 eyes (10.5%), a large submacular haemorrhage was present in 2 eyes (5.2%), and a disciform scars was present in 5 eyes (13.1%) (Table 1).

### 3.2. Visual Outcomes

**Figure 1:**
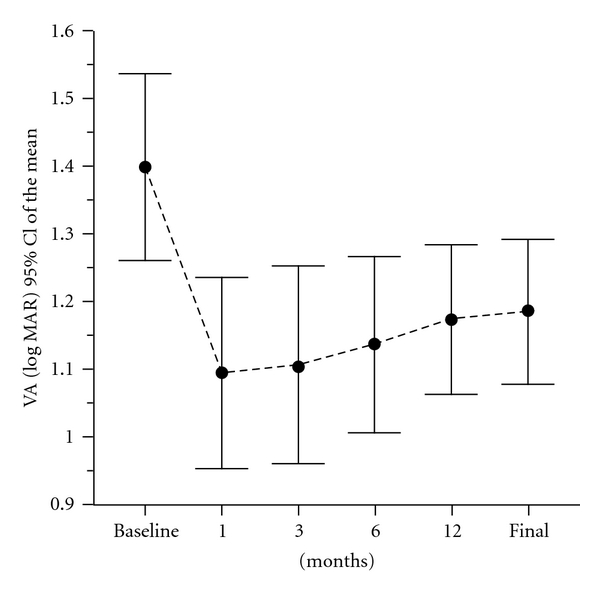
Evolution of change in logMar visual acuity over 14 months after intravitreal injection of bevacizumab showing statistically significant improvement.

The mean visual acuity (VA) at baseline was 20/400 (logMAR 1–2, mean logMAR 1.38 ± 0.33) (range hand movements to 20/200). VA was less than 20/200 in 30 patients (78.9%) and equal to 20/200 in 8 patients (21%). Mean BCVA improved to 20/250 (1.17 logMAR), and this difference was statistically significant (*P* < 0.001) (Figure 1). At final examination, 11 eyes (28.9%) showed stable vision and only 3 eyes (8%) experienced visual acuity worsening. Visual acuity improved in 24 eyes (63.2%) by 3 lines or more in 18 eyes (47.3%) with a mean gain of 2.32 lines. Metamorphopsia, blurred vision, and scotoma were consistently reduced inducing the improvement of quality of vision in all cases.

Analysis of visual acuity in patients previously treated with PDT (*n* = 10) and patients without prior PDT (*n* = 28) show that both groups benefited from bevacizumab treatment (Table 2). Visual acuity improved, respectively, from 1.3 logMAR ± 0.24 to 1.16 logMAR ± 0.15 with a statistically limit significant difference (*P* = 0.043; paired Wilcoxon test) in patients previously treated with PDT and from 1.4 logMAR ± 0.36 to 1.18 logMAR ± 0.38 with statistically significant difference (*P* = 0.001; paired Wilcoxon test) in non-PDT patients. Moreover the visual acuity improved by 3 lines or more in 15 eyes (53.57%) in patients without prior PDT compared with only 3 eyes (30%) in patients with prior PDT.

### 3.3. Visual Acuity Analysis

At one month, mean BCVA improved to 1.12 logMAR ± 0.37 (*P* = 10^−4^, *n* = 37, paired Student's test). At 3 months, mean BCVA remained stable to 1.11 logMAR ± 0.36 (P = 10^−4^, paired Student's test). At the 6 month follow-up examination, mean BCVA was 1.14 logMAR ± 0.37 (*P* = 0.001, paired Student's test), mean gain was 2.15 lines ± 3.45. At the 9-month time point, mean BCVA was 1.19 logMAR ± 0.36 (*P* = 0.003, paired Wilcoxon test), mean gain was 2 lines ± 3.41. At 12 months, the mean BCVA decreased slightly to 1.22 logMar ± 0.33 but compared to baseline difference was also statistically significant (*P* = 0.004, paired Wilcoxon test). Mean gain was 1.7 lines ± 3.07. VA improved at least by three lines in 48%, was stable in 47.3%, and deteriorates in one patient. If we consider at 12 months only eyes without prior PDT treatment, visual acuity was 1.28 logMAR ± 0.37 with a statistically significant difference (*P* = 0.033).

### 3.4. Status of Exudative Changes (Figure 2)

**Figure 2:**
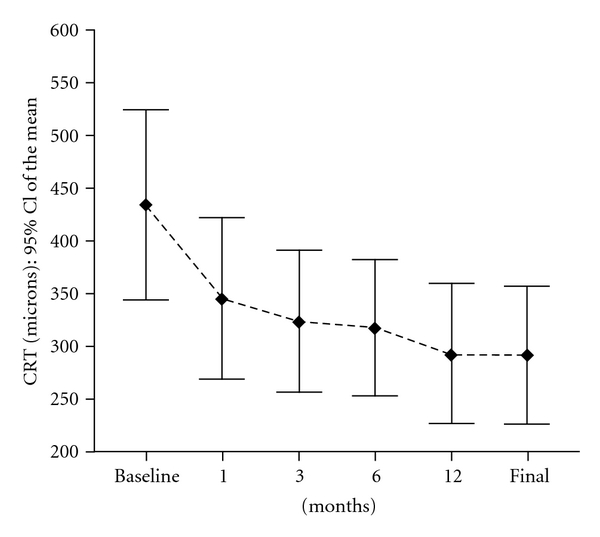
Evolution of change in retinal thickness over 14 months after intravitreal injection of bevacizumab showing statistically significant improvement.

Average CRT was 431 microns ± 159.7 (range 211–965) at baseline. The CRT decreased statistically to 304.61 *μ*m ± 133.36 (range 123–620 *μ*m) (*P* < 0.001) (Figure 2).

The CRT decreased to 334.67 *μ*m ± 137.14 at one month, to 318.67 *μ*m ± 124.12 at three months, to 293.43 *μ*m ± 122.79 at six months, and to 294.23 *μ*m ± 123.8 at nine months (*P* = 10^−4^, paired Wilcoxon test). At 12 months, the Mean central retinal thickness remained stable to 293.1 *μ*m ± 130 (*P* = 0.004, paired Wilcoxon test).

CRT decreased, respectively, from 472.33 *μ*m ± 61.23 to 323.4 *μ*m ± 159.6 (*P* = 0.1; paired Wilcoxon test) in patients previously treated with PDT (*n* = 10) and from 427.11 *μ*m ± 167.52 to 301 *μ*m ± 131.08 with a statistically significant difference (*P* = 10^−4^; paired Wilcoxon test) in patients without prior PDT (*n* = 28). In this group, at 12 months mean CRT was 313.31 *μ*m ± 138.9 and the difference was maintained statistically significant (*P* = 0.01; paired Wilcoxon test) (Table 2).

### 3.5. Retreatment

Patients received a mean of 2.86 ± 0.77 IVB (range 2–5 injections). Twenty-one (55.2%) eyes received three injections, 12 eyes (31.5%) received less than three injections, and only five eyes (13.1%) received more than three injections.

### 3.6. Adverse Effects

A total of 109 injections were performed. One case of retinal detachment had occurred. No other ocular or systemic complications appeared during followup.

## 4. Discussion

Early treatment of wet AMD may limit the CNV-induced damage to the photoreceptors or the retinal pigment epithelium, leading to a better visual acuity outcome. Unfortunately, many patients are not diagnosed early nor have chronic disease after receiving other treatment modalities with low visual acuity [4, 5].

Our findings revealed in patients with low visual acuity a statistically significant improvement in visual acuity associated with reduced foveal thickness on OCT after bevacizumab treatment. These findings compare well with other series in the literature [5, 6]. Ehrlich et al. [5] and Galbinur et al. report similar results, respectively, at 27 weeks and 3.9 months mean followup; here, we demonstrate that visual improvement can last 6 and even 12 months after treatment.

In all included patients, VA was measured with the ETDRS chart which is more accurate, in the lower VA range. As patients do not have good fixation, there was a learning curve for measurements of visual acuity and, moreover, OCT has been more difficult and longer to implement. So, several scans were often needed to measure the central retinal thickness and localize the fovea.

In our report, at 12 months, visual improvement occurred in 48% of eyes (13/27) at least by three lines with mean gain of 1.7 lines ± 3.07; stabilisation occurred in 48% (13/27). The learning curve may affect the improvement of visual acuity after the treatment.

Overall, improvement in quality of vision, especially the decrease of metamorphopsia, made the patient's daily life easier. Only one patient showed deterioration of her VA from 1.13 logMAR to 1.1 logMAR. There was a greater improvement of VA in patients with no atrophic or fibrotic changes, who were not treated previously with PDT and in patients with smaller size lesions.

Our study includes only ten patients with previous PDT treatment. It is noteworthy that patients who were not treated previously with PDT benefited more from intravitreal bevacizumab compared with those who underwent prior PDT due to possible damage caused by multiple PDT sessions [5, 7]. We found a statistically significant difference in response between patients who had received PDT treatment and those who had not and this is in accordance with the report of Ehrlich et al. [5].

Visual improvement by three lines or more was found in 48% of our patients and in 53.5% if we consider only patients not treated with prior PDT; this finding is similar to that of Galbinur et al. [6] who reported an improvement of 43%, however, in the report of Ehrlich et al. [5], the same improvement is limited to only 25%. The fact that the majority of patients (66%) had previously received photodynamic therapy while none of the patients of those treated by Galbinur et al. [6] and only 26.3% of our patients had previous photodynamic therapy.

In our study, the mean central retinal thickness was significantly reduced from 404 *μ*m to 291.50 *μ*m (*P* < 0.001). Significant decrease in central retinal thickness was reported by Ehrlich et al. [5] measuring at baseline 324 ± 121 *μ*m to 264 ± 65 *μ*m at final examination (*P* = 0.02). We assumed that a decrease in the leakage and absorption of the subretinal and intraretinal fluid would improve the function of the remaining viable photoreceptors.

In accordance with previous studies, significant decrease in central retinal thickness was seen from the first to the third month, where mean macular thickness decreased from 431 *μ*m ± 159.7 to 334.67 *μ*m ± 137.14 at one month and to 318.67 *μ*m ± 124.12 at 3 months showing rapid effect during treatment. From 6 to 12 months, macular thickness remained stable.

Our data indicate that intravitreal bevacizumab injection results in a significant improvement in functional and anatomic outcomes from the first month after injection, maintained at 12 months after treatment. In the absence of subfoveal fibrosis, intravitreal bevacizumab injection is beneficial in patients with neovascular AMD and severe vision loss. Treatment decisions should not be based on the visual acuity but on the characteristics of the lesion.

The strength of our study is a long follow-up period, use of standardized ETDRS protocol visual acuity data and acquisition of OCT data at each follow-up visit and by the same investigator. Limitation of this study includes its retrospective design with lack of a control group.

Our results suggest that intravitreal bevacizumab is well tolerated and may increase the chance of visual acuity gain in neovascular age-related macular degeneration even in cases with initial low vision. So, this study supports the findings of previously published series regarding the short-term improvement in visual acuity and central retinal thickness but suggests 6- and 12-month improvement. Given the high percentage of patients who present with advanced AMD, larger controlled trials are warranted.

## Figures and Tables

**Table 1:** Baseline features of patients with age-related macular degeneration and initial low visual acuity.

Mean age + SD	73 + 6.24
Mean logMar VA	1.38
Mean greatest linear dimension (*μ*)	1748,02
Mean central retinal thickness (*μ*m)	431 + 159.7 (range, 211–965)

No prior PDT	73.6%
Angiographic characteristic	
Predominantly classic or classic	22 (28.9%)
Predominantly occult or occult	54 (71.05%)
CNV associated to Subretinal hemorrhage	4 (5.2%)
CNV associated to pigment epithelial detachment	8 (10.5%)

LogMAR: logarithm of the minimum angle of resolution; VA: visual acuity; OCT: optical coherence tomography; CNV: choroidal neovascularisation

**Table 2:** Visual acuity (VA) and central retinal thickness (CRT) outcome in patients previously treated with PDT compared with patients without prior PDT.

	Patients with prior PDT (*n* = 10)	*P*	Patients without prior PDT (*n* = 28)	*P *
VA(logMAR)				
(i) At baseline	1.3 ± 0.24		1.4 ± 0.36	
(ii) At final examination	1.16 ± 0.15	0.043	1.18 ± 0.38	0.001
(iii) At 12 months	1.08 ± 0.2	0.039	1.28 ± 0.37	0.03

CRT (*μ*m)				
(i) At baseline	472.33 ± 61.23		427.11 ± 167.52	
(ii) At final examination	323.4 ± 159.6	0.1	301 ± 131.08	10^−4^
(iii) At 12 months	312.75 ± 184.9	0.18	313.31 ± 138.9	0.01

LogMAR: logarithm of the minimum angle of resolution; VA: visual acuity; CRT: central retinal thickness; PDT: photodynamic therapy.
